# Unraveling the role of bacteria with heritable versus non-heritable relative abundance in the gut on boar semen quality

**DOI:** 10.1186/s12711-025-00990-2

**Published:** 2025-11-06

**Authors:** Liangliang Guo, Xiaoqi Pei, Jiajian Tan, Haiqing Sun, Siwen Jiang, Hongkui Wei, Jian Peng

**Affiliations:** 1 Department of Animal Nutrition and Feed Science, College of Animal Science and Technology, Huazhong Agricultural University, Wuhan, 430070 China; 2 YangXiang Joint Stock Company, Guigang, 537000 China; 3 Key Lab of Agricultural Animal Genetics, Breeding and Reproduction of Ministry of Education & Key Lab of Swine Genetics and Breeding of Ministry of Agriculture, Huazhong Agricultural University, Wuhan, 430070 China; 4 The Cooperative Innovation Center for Sustainable Pig Production, Wuhan, 430070 China; 5 Frontiers Science Center for Animal Breeding and Sustainable Production, Wuhan, 430070 China

## Abstract

**Background:**

The relative abundance of some bacteria in the gut of pigs is heritable, suggesting that host genetics may recursively influence boar semen quality by affecting the composition and function of gut microbiota. Therefore, it is essential to elucidate the specific contributions of heritable versus non-heritable gut microbiota to semen quality traits.

**Results:**

Our study aimed to identify heritable and non-heritable bacterial taxa at the genus level in the boar gut and to predict their functions and respective contributions to semen quality traits. At the genus level, 39 heritable and 91 non-heritable bacterial taxa were identified. Functional analysis revealed that predicted microbial functions in both groups were primarily enriched in carbohydrate, nucleotide, and amino acid metabolism. We further analyzed the average microbiability of heritable and non-heritable bacteria on short-chain fatty acids (SCFAs) and semen quality traits. The relative abundance of heritable bacteria was found to contribute more to SCFAs levels and semen quality than non-heritable bacteria. Mediation analysis revealed that SCFAs could mediate the influence of the relative abundance of heritable bacteria on host phenotypes, identifying 99 significant genus-SCFAs-semen quality trait mediation links.

**Conclusions:**

Our findings underscore the substantial role of the relative abundance of heritable gut bacteria in shaping porcine semen quality through SCFAs mediation. These results highlight the potential of targeted microbiome interventions to enhance reproductive traits in pigs.

**Supplementary Information:**

The online version contains supplementary material available at 10.1186/s12711-025-00990-2.

## Background

The porcine gut microbiome harbors a gene pool vastly exceeding that of the host and is widely recognized as the host’s “second genome” due to its symbiotic relationship with the host [1]. Short-chain fatty acids (SCFAs) are key metabolic products synthesized by the gut microbiota through the fermentation of dietary fiber [2]. SCFAs, including acetate, propionate, and butyrate, play a crucial role in maintaining gut health and exert systemic effects on the host’s metabolism and immune function [3, 4]. The influence of SCFAs extends to reproductive health, with numerous studies demonstrating their regulatory role in semen quality traits in pigs [5–7]. Therefore, the gut microbiota and the SCFAs they synthesize constitute promising targets for improving pig semen quality.

While the prevailing consensus has long attributed the shaping of gut microbiota primarily to dietary and environmental factors [8], recent studies have shown that significant differences still exist in the gut microbiota of animals, even when external factors such as diet and environment are consistent [9–11]. Notably, the genetic backgrounds of the animals in these studies exhibited variation, strongly suggesting the influence of host genetics on the gut microbiota. Significant variations in the gut microbiota have been observed among pig breeds [9, 11]. Moreover, emerging evidence from quantitative genetics, particularly genome-wide association studies, has shed new light on the substantial impact of host genetics in shaping the gut microbiota structure in pigs [12, 13].

The heritability (h^2^) of the relative abundance of gut microbiota can be used to distinguish between heritable and non-heritable bacteria. Researchers have developed the concept of microbiability (m^2^), adapting heritability estimation methods [14]. Microbiability reflects the extent to which host phenotypes are influenced by gut microbiota. Khanal et al. showed that gut microbiota composition has a significant effect on meat quality and carcass traits in commercial pigs, with the microbiability of these traits increasing with age [15]. Similarly, Aliakbari et al. reported that the microbiability of residual feed intake, feed conversion efficiency, daily feed intake, average daily gain, and backfat thickness in Yorkshire pigs ranges from 0.02 to 0.20 [16]. These studies suggest that certain phenotypes in pigs are significantly influenced by gut microbiota composition and the magnitude of this influence can be estimated using microbiability. However, current research has primarily focused on growth and carcass traits, leaving the microbiability of semen quality traits in boars largely unexplored. Furthermore, existing studies have estimated microbiability using the entire gut microbiota without distinguishing between heritable and non-heritable bacteria.

Accordingly, this study sought to characterize heritable and non-heritable bacterial genera by estimating the heritability of their relative abundance, based on 16S rRNA gene sequencing and host genotyping data. This study investigates the functional differences between these two categories of gut bacteria and determines their microbiability for boar semen quality traits. Additionally, this study explores the mediating role of SCFAs in the relationship between heritable bacteria and host phenotypes.

## Methods

### Experimental animals and sample collection

A total of 556 boars from three breeds, including purebred Duroc (n = 175), Landrace (n = 195), and Yorkshire (n = 186), were raised under uniform conditions at an artificial insemination station in southern China. These boars were between 8 and 69 months of age. The boar-rearing conditions followed previous descriptions [5]. Briefly, boars were housed individually in pens within a barn equipped with negative-pressure ventilation. The barn had 12-h light and dark cycles. Boars received approximately 2.5 kg of feed per day and had free access to water; the diet composition is detailed in Additional file 1: Table S1.

Fresh feces were collected from all 556 boars via rectal massage, and whole blood samples were drawn from the hindlimb veins of 552 boars (n = 172 for Duroc, n = 195 for Landrace, and n = 185 for Yorkshire) using blood collection tubes. Blood samples were stored at − 20 °C, and fecal samples were flash-frozen in liquid nitrogen immediately after collection and subsequently stored at − 80 °C. Additionally, the short-chain fatty acid content in feces was determined using gas chromatography, following previous methods [5].

Semen was collected from each boar approximately every 5 days, and semen quality was analyzed at each collection, including semen volume, sperm concentration, sperm motility, and abnormal sperm rates. These parameters were measured as previously described [5]. To prevent single test errors, the mean semen parameter values were calculated from semen data collected 1 month before and 1 month after the feces sampling point. This value reflects the semen quality of the boar over a recent period of time.

### 16S rRNA sequencing

Total microbial DNA was extracted from approximately 2 g of fecal sample from each boar using the QIAamp Fast DNA Stool Kit (Qiagen, Germany). DNA was quantified using a Nanodrop and quality was assessed through 1.2% agarose gel electrophoresis. The V3–V4 regions of the 16S rRNA gene were amplified using the forward primer 341F (ACTCCTACGGGAGGCAGCA) and reverse primer 806R (GGACTACHVGGGTWTCTAAT). The amplified products were purified using magnetic beads (Vazyme VAHTSTM) and subjected to fluorescence quantification (BioTek FLx800) to adjust sample ratios. Sequencing libraries were prepared using Illumina’s TruSeq Nano DNA LT Library Prep Kit, and paired-end sequencing was performed on a NovaSeq sequencer (Shanghai Personal Biotechnology Co., Ltd.). Downstream sequencing data underwent quality control and were analyzed using the QIIME2 DADA2 (2019.4) pipeline for sequence clustering [17]. Amplicon sequence variant (ASV) taxonomy was assigned through a BLAST search against the Silva database (version 132) [18]. An ASV table was generated to record the abundance and classification of each ASV per sample. The ASV table was flattened, and distance matrices for each sample were computed. A range of unsupervised sorting and clustering methods was used to quantify variations in beta diversity and assess the significance of differences between samples (or groups), along with relevant statistical tests.

### Genotyping

Genomic DNA was extracted from the 552 boar blood samples and genotyped using a 50K SNP Beadchip (comprising 51,368 SNPs, Beijing Compass Biotechnology Co., Ltd). SNP quality control was performed using Plink1.9 software [19] based on the following criteria: (1) SNPs at unknown locations and on sex chromosomes were excluded, (2) individuals with SNP call rates < 90% were excluded, (3) SNPs with call rates < 90% were excluded, and (4) SNPs with minor allele frequencies < 5% were excluded. After quality control, missing genotypes were imputed using Beagle software [20], followed by a second round of quality control. In total, 34,235 SNPs from 552 individuals were retained.

### ***Estimation of the heritability (h***^***2***^***) and total microbiability (m***^***2***^***) of SCFAs and semen quality traits***

Five hundred fifty-two boars with both genotype and microbial profile data were included in the analysis. The genomic relationship matrix (**G**) was constructed across breeds using the Hiblup software [21]. The microbiome relationship matrix (**M**) was built based on the relative abundance of 1027 ASVs that were detected in more than 30% of individuals. The construction method of **M** was described by Khanal et al. [22]. In short, we first construct relative abundance matrices **S** (**n** × **v**), where **n** is the number of animals, and **v** is the number of ASVs. The relative abundance matrix **S** was log-transformed after adding 0.00001 and then normalized using Z-score normalization to produce the elements of the **H** matrix as:

1 $$ {\text{H}}_{{{\text{ij}}}} = \frac{{\log \left( {{\text{S}}_{{{\text{ij}}}} } \right) - \overline{{\log \left( {{\text{S}}_{{.{\text{j}}}} } \right)}} }}{{{\text{sd}}\left( {\log \left( {{\text{S}}_{{.{\text{j}}}} } \right)} \right)}} $$

where **S**_**.j**_ is the **j**-th column vector of matrix **S**. The term sd stands for standard deviation. The **H** matrix represents the log-transformed, centered, and standardized relative abundance of ASVs. The **M** matrix was calculated as: $\mathbf{M}=\frac{1}{\mathbf{v}}\mathbf{H}{\mathbf{H}}^{\mathbf{T}}$**.**

To estimate the h^2^ and total m^2^ of semen traits and SCFA concentrations, we employed a linear mixed model implemented via the Hiblup software using the genomic restricted maximum likelihood (GREML) method, using the following model:

2 $$ {\mathbf{y}} = {\mathbf{Ac}} + {\mathbf{g}} + {\mathbf{b}} + {\mathbf{e}}. $$

where **y** represents the vector of phenotypes, and **c** is the vector of fixed effects, including breed and age (as a covariate), **A** is the corresponding incidence matrix, **e** represents the vector of residuals, and **g** is the vector of host genetic effects based on all SNPs. The distribution of **g** follows **N(0,**${\mathbf{G}{\varvec{\upsigma}}}_{\mathbf{A}}^{2}$**)**, where **G** represents the matrix of host-genome relationships and ${{\varvec{\upsigma}}}_{\mathbf{A}}^{2}$ denotes the additive genetic variance. Vector **b** represents the collective effect of all ASVs, distributed as **N(0,**${\mathbf{M}{\varvec{\upsigma}}}_{\mathbf{b}}^{2}$**)**, where **M** denotes the microbiome relationship matrix, and ${{\varvec{\upsigma}}}_{\mathbf{b}}^{2}$ is the additive microbiome variance.

### ***Estimation of the h***^***2***^*** of gut microbes***

Data on bacterial genera and ASVs were first filtered by prevalence. Genera with a prevalence > 60% and ASVs with a prevalence > 30% were selected. To approximate a normal distribution prior to analysis, microbial relative abundances were subjected to centered log-ratio transformation. SNP heritability was estimated separately for each bacterial genus and ASV using the GREML algorithm in the GCTA software [23]. The top five host genetic principal components based on SNP genotypes were included as covariates to adjust for population structure. Additional factors potentially affecting the boar gut microbiota were included as fixed effects in the following model used to estimate heritability:

3 $$ {\mathbf{y}} = {\mathbf{Kc}} + {\mathbf{g}} + {\mathbf{e}}. $$

where **y** represents the vector of phenotypes, **c** is the vector of fixed effects (the first five host genetic components, breed, and months of age as a covariate), and **K** is the corresponding incidence matrix. The contents of **g** and **e** are consistent with those described above. Heritability estimates of the relative abundance of the genera were visualized by using the iTOL tool [24].

### ***Estimation of the m***^***2***^*** of heritable and non-heritable bacterial for SCFAs and semen quality traits***

Here, the relationship among heritable and non-heritable microbiota was captured by constructing microbiability relationship matrices **M**_**1**_ and **M**_**2**_, respectively. We first constructed relative abundance matrices **S**_**1**_ (**n** × **p**, for heritable ASVs) and **S**_**2**_ (**n** × **q**, for non-heritable ASVs), where **n** is the number of animals, **p** is the number of heritable ASVs, and **q** is the number of non-heritable ASVs, which were then used to construct **H**_**1**_ and **H**_**2**_ according to the method described above. The heritable bacteria relationship matrix **M**_**1**_ was calculated as: ${\mathbf{M}}_{1}=\frac{1}{\mathbf{p}}{\mathbf{H}}_{1}{\mathbf{H}}_{1}^{\mathbf{T}}$. Similarly, the non-heritable bacteria relationship matrix **M**_**2**_ was calculated as: ${\mathbf{M}}_{2}=\frac{1}{\mathbf{q}}{\mathbf{H}}_{2}{\mathbf{H}}_{2}^{\mathbf{T}}$. Using these relationship matrices (**M**_**1**_ and **M**_**2**_), the contribution of heritable and non-heritable microbiota to host phenotypes was estimated using the following model, separately for the heritable and non-heritable microbiota:

4 $$ {\mathbf{y}} = {\mathbf{Xc}} + {\mathbf{b}} + {\mathbf{e}}. $$

where **y** represents the vector of phenotypes, including semen parameters and SCFAs. **c** is the vector of fixed effects (breed and months of age as a covariate), **X** is the corresponding incidence matrix, **e** represents the residuals, and **b** represents the combined effects of all included ASVs, distributed as **N (0,** ${\mathbf{M}{\varvec{\upsigma}}}_{\mathbf{b}}^{2}$**)**, where **M** denotes the microbiome relationship matrix, and ${{\varvec{\upsigma}}}_{\mathbf{b}}^{2}$ is the additive microbiome variance.

To account for the differing numbers of heritable and non-heritable bacteria, the average contribution of the relative abundance of individual taxa to SCFAs and semen quality traits was estimated using average microbiability [25].

5 $$ {{\varvec{\upbeta}}}_{{\mathbf{1}}}^{{\mathbf{2}}} = \frac{{{\mathbf{m}}_{{\mathbf{1}}}^{{\mathbf{2}}} }}{{\mathbf{p}}}. $$

6 $$ {{\varvec{\upbeta}}}_{{\mathbf{2}}}^{{\mathbf{2}}} = \frac{{{\mathbf{m}}_{{\mathbf{2}}}^{{\mathbf{2}}} }}{{\mathbf{q}}}. $$

where **β**^**2**^ represents the average microbiability of individual taxa, **m**^**2**^ is the overall microbiability (the contribution of heritable and non-heritable microbiota), **p** is the number of heritable ASVs, and **q** is the number of non-heritable ASVs. The microbiability of heritable microbes is denoted by ${\mathbf{m}}_{\mathbf{h}}^{2}$, and that of non-heritable microbes is denoted by ${\mathbf{m}}_{\mathbf{n}\mathbf{o}\mathbf{n}}^{2}$.

### Functional prediction of heritable and non-heritable bacteria

The 16S rRNA sequences of heritable and non-heritable ASVs were used to predict microbial function using the PICRUSt2 tool (https://github.com/picrust/picrust2/wiki). The functional predictions were compared with pathways in the MetaCyc database (https://metacyc.org) to assess the metabolic capabilities of the heritable and non-heritable microbes.

### Mediation linkage inference

To explore potential correlations between the relative abundance of heritable bacteria and semen quality phenotypes, Spearman correlation analysis was performed to retain only heritable bacteria whose relative abundance was significantly associated with at least one semen quality trait (*P* < 0.05). Mediation analysis was performed using the mediate function from the R package mediation (version 4.5.0) to evaluate the potential causal influence of gut microbiota (vector of independent variables **x**) on host phenotypes (**y**) through short-chain fatty acids (vector of SCFAs as mediators, **mediator**). Two linear regression models were constructed: a mediator model (**mediator ~ x + age**) and an outcome model (**y ~ x + mediator + age**), with **age** included as a covariate in both models to control for potential confounding. The mediate function was applied with nonparametric bootstrapping using 1000 simulations to estimate the average causal mediation effect and average direct effect, along with their 95% confidence intervals.

## Results

### Boars of different breeds exhibit significant differences in semen quality

**Fig. 1:**
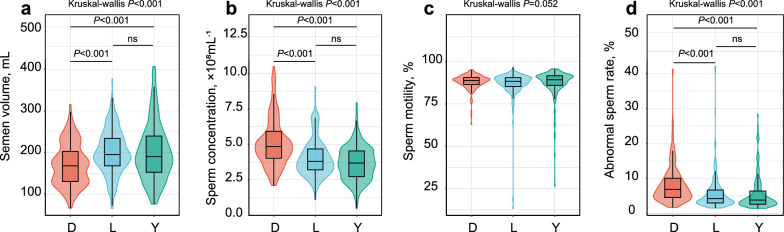
Significant differences in semen quality between breeds. **a** Semen volume. **b** Sperm concentration. **c** Sperm motility. **d** Abnormal sperm rate. Differences between groups were compared using the Kruskal‒Wallis rank sum test. Once the Kruskal–Wallis test found significant differences (*P* < 0.05), multiple comparisons were made using Dunn’s Test. *D* Duroc, *L* Landrace, *Y* Yorkshire

We analyzed semen quality traits of 552 boars (n = 172 for Duroc, n = 195 for Landrace, and n = 185 for Yorkshire) using both microbiome and genomic data. Significant differences in semen quality were observed across the three breeds (Fig. 1). Duroc boars had significantly lower semen volumes compared to Landrace and Yorkshire boars (*P* < 0.001, Fig. 1a). Conversely, sperm concentration was significantly higher in Duroc boars than in the other two breeds (*P* < 0.001, Fig. 1b). Sperm motility did not significantly differ between the three breeds (*P* = 0.052, Fig. 1c). However, Duroc boars had a significantly higher of abnormal sperm rate compared to Landrace and Yorkshire boars (*P* < 0.001, Fig. 1d).

### Gut microbiota composition differs between breeds

**Fig. 2:**
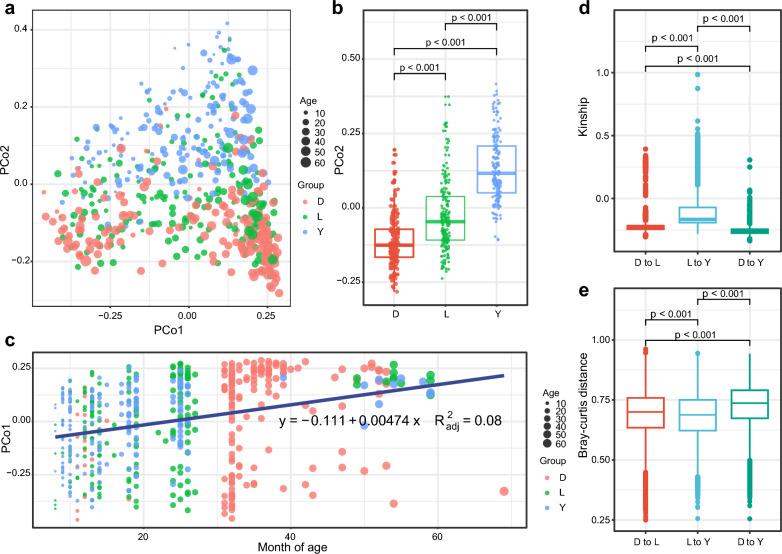
Breeds drive variation in boar gut microbiota. **a** Principal coordinates analysis plot based on Bray–Curtis distances. **b** Projected distance of samples along the PCo2 axis. **c** Projected distance of boars along the PCo1 axis. **d** Kinship between breeds. **e** Microbial distance between breeds. Differences between groups were compared using the Kruskal‒Wallis rank sum test; when differences were significant, multiple comparisons were made using Dunn’s test. The age of pigs was expressed in months. Linear regression was utilized to establish the relationship between age and the PCo1 index. *D* Duroc, *L* Landrace, *Y* Yorkshire

The gut bacterial profiles from 556 boars were analyzed using amplicon sequencing. Initial analysis revealed an average of 55,347 quality-filtered bacterial sequences per boar. After denoising and clustering, 45,232 ASVs were identified, spanning 42 phyla, 96 classes, 217 orders, 399 families, and 1040 genera. To explore potential breed-associated patterns in microbial communities, we compared the gut microbiota composition across Duroc, Landrace, and Yorkshire boars. Principal coordinates analysis based on Bray–Curtis distances showed significant separation of gut microbiota between breeds along the PCo2 axis, indicating a substantial impact of breed on gut microbiota composition (Fig. 2a). Further analysis demonstrated significant differences in the projection distances of samples along the PCo2 axis between the three breeds (Fig. 2b). Additionally, we found that differences along the PCo1 axis were related to the age of the boars, as projection distances increased linearly with age (Fig. 2c).

Using SNP chip data, we assessed the kinship among boars and found that the kinships between Duroc and Yorkshire boars were significantly lower than those between Duroc and Landrace boars or between Landrace and Yorkshire boars (Fig. 2d). Further analysis of Bray–Curtis distances between boars with different kinships showed that the microbial distance between Duroc and Yorkshire boars was significantly greater than between Duroc and Landrace boars or between Landrace and Yorkshire boars (Fig. 2e). Thus, boar breeds with more distant kinships also exhibited more pronounced differences in gut microbiota composition, suggesting that host genetic factors play a crucial role in shaping the gut microbiota structure.

### Identification of heritable and non-heritable taxa in the gut microbiota of boars

**Fig. 3:**
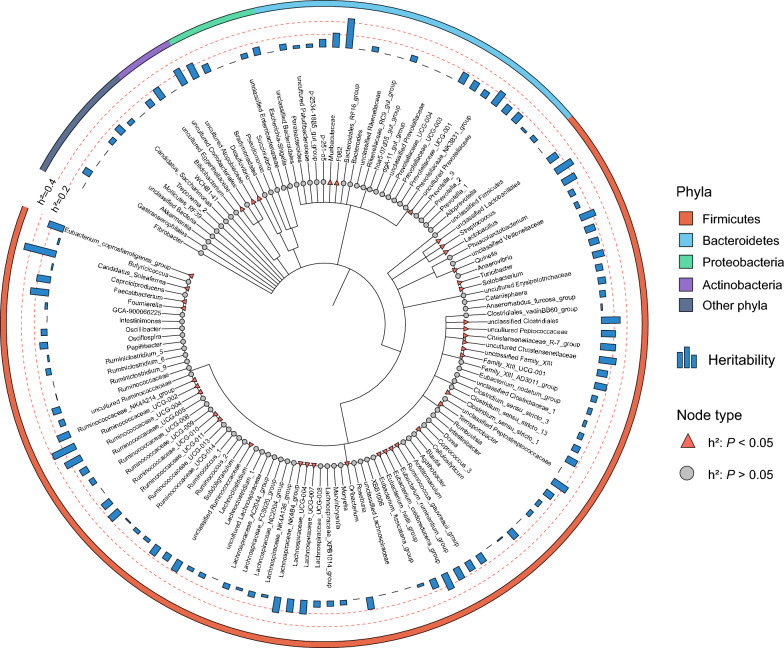
Identification of genera with heritable relative abundance in the boar gut. From the inside to the outside, the figure displays the phylogenetic tree of genera, the bar chart of heritability estimates, and the corresponding phyla. Genera with significant heritability are marked with red triangles (*P* < 0.05), while genera with non-significant heritability are marked with gray circles (*P* > 0.05). The tree was created using the iTOL (https://itol.embl.de)

In this study, we performed genotyping analysis on 552 boars using the Porcine 50K SNP Beadchip (comprising 51,368 SNPs). Following genotype imputation and quality control, a dataset comprising 34,235 SNPs was utilized to generate the genetic relatedness matrix **G**. Population structure analysis indicated genetic differentiation between the three boar breeds (see Additional file 2: Figure S1). Given that breed differences reflect underlying genetic variation, our previous results also indicate that breed is a major determinant of gut microbiota composition in boars (Fig. 2). To further explore host genetic effects on the gut microbiota, we estimated the heritability of the relative abundance of 130 genera (present in ≥ 60% of samples) using the GREML algorithm implemented in GCTA. Of these common 130 genera, 39 showed significant heritability of their relative abundance (*P* < 0.05), while 91 did not (Fig. 3 and see Additional file 1: Table S2). These heritable bacteria were distributed across four phyla: Firmicutes (33), Bacteroidetes (3), Actinobacteria (2) and Proteobacteria (1) (Fig. 3). Among the heritable microbes, 23 taxa had h^2^ > 0.2, including two with high heritability (h^2^ > 0.4) (Fig. 3). Some taxa within the Firmicutes phylum showed high heritability estimates, including *Candidatus_Soleaferrea* (h^2^ = 0.51 ± 0.09, mean ± SE), *Ruminococcaceae_UCG-002* (h^2^ = 0.39 ± 0.10), and *Streptococcus* (h^2^ = 0.30 ± 0.10). A member of the Bacteroidetes phylum, *F082*, also exhibited a high heritability estimate (h^2^ = 0.46 ± 0.10).

### Functional predictions of heritable and non-heritable bacteria

**Fig. 4:**
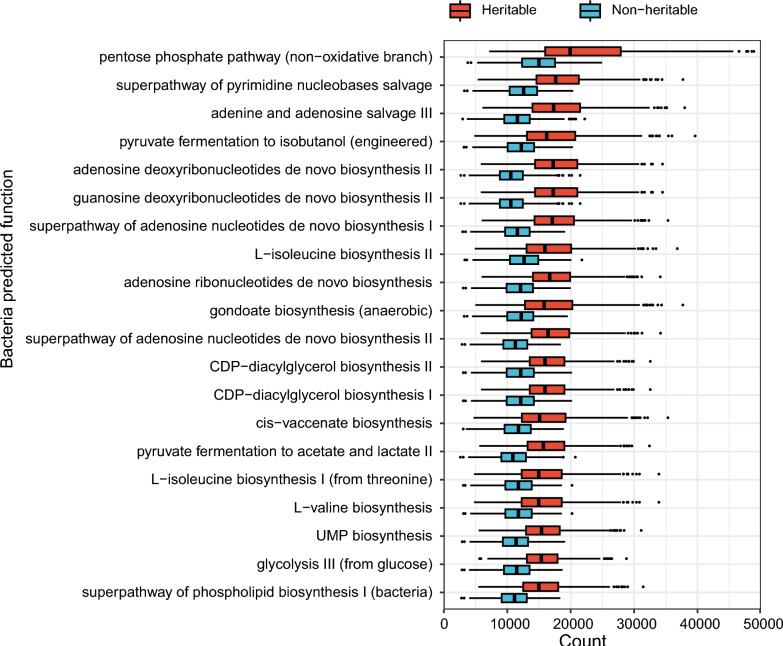
Prediction of the function of bacteria with heritable and non-heritable relative abundance in the boar gut

To further explore the functional characteristics of heritable and non-heritable bacteria, we used the PICRUSt2 tool for microbial functional prediction [26]. Since this tool operates only at the ASV level, we first estimated the heritability of 1027 ASVs with a presence rate greater than 30%, classifying them into 352 heritable and 675 non-heritable ASVs (see Additional file 1: Table S3). Functional prediction was then performed separately for the two groups to reveal potential functional differences associated with host genetic influences on the gut microbiota. In total, 268 predicted biochemical functions were identified in the heritable bacteria and 316 in the non-heritable bacteria. To explore the main functional differences between heritable and non-heritable bacteria, we selected and compared the top 20 most abundant predicted functions [25]. These top 20 functions included pathways primarily associated with carbohydrate metabolism (e.g., Pentose phosphate pathway, Glycolysis, Pyruvate fermentation to isobutanol, and Pyruvate fermentation to acetate and lactate), nucleotide synthesis and salvage pathways (e.g., Superpathway of pyrimidine nucleobases salvage, Adenine and adenosine salvage, Adenosine deoxyribonucleotides and Guanosine deoxyribonucleotides de novo biosynthesis, Adenosine ribonucleotides de novo biosynthesis, etc.), and amino acid and lipid synthesis pathways (e.g., l-isoleucine biosynthesis, l-valine biosynthesis, Gondoate biosynthesis, Cis-vaccenate biosynthesis, Superpathway of phospholipid biosynthesis I, and CDP-diacylglycerol biosynthesis, etc.) (Fig. 4). Notably, these functions were more enriched among heritable bacteria than among non-heritable bacteria, suggesting a stronger role of heritable taxa in essential metabolic and biosynthetic processes (Fig. 4).

### Quantifying the effects of host genetics and gut microbiota (heritable and non-heritable) on phenotypic traits

To explore the contributions of host genetics, total microbiota, heritable microbes, and non-heritable microbes to SCFAs levels and semen phenotypes, we constructed a series of linear mixed models to estimate trait **h**^**2**^, total **m**^**2**^, heritable microbes **m**^**2**^ (${\mathbf{m}}_{\mathbf{h}}^{2}$), and non-heritable microbes **m**^**2**^ (${\mathbf{m}}_{\mathbf{n}\mathbf{o}\mathbf{n}}^{2}$). Table 1 showed that for SCFA traits, total **m**^**2**^ was generally higher than **h**^**2**^, whereas for semen traits, **h**^**2**^ exceeded total **m**^**2**^. Notably, for SCFAs, the ${\mathbf{m}}_{\mathbf{n}\mathbf{o}\mathbf{n}}^{2}$ was larger than ${\mathbf{m}}_{\mathbf{h}}^{2}$. However, on average, heritable microbiota explained more variance per ASV than non-heritable ones. In contrast, for semen traits, the **m**^**2**^ estimates of heritable and non-heritable microbiota were relatively similar. In particular, the estimates for sperm motility and abnormal sperm rate were negligible. Nonetheless, similar to the pattern observed for SCFAs, the average microbiability of heritable microbes remained numerically higher than that of non-heritable microbes for semen traits (Table 1).

**Table 1:** Estimates of the **h**^**2**^, total **m**^**2**^, ${\mathbf{m}}_{\mathbf{h}}^{2}$ and ${\mathbf{m}}_{\mathbf{n}\mathbf{o}\mathbf{n}}^{2}$ of phenotypes

**Traits**	**h**^**2**^	**Total m**^**2**^	${\mathbf{m}}_{\mathbf{h}}^{2}$	${\mathbf{m}}_{\mathbf{n}\mathbf{o}\mathbf{n}}^{2}$
Acetate	0.00 ± 0.09	0.26 ± 0.07	0.00 ± 0.03	0.10 ± 0.06
Propionate	0.15 ± 0.08	0.38 ± 0.06	0.35 ± 0.06	0.51 ± 0.06
Butyrate	0.10 ± 0.07	0.51 ± 0.05	0.46 ± 0.05	0.63 ± 0.05
Valerate	0.05 ± 0.07	0.44 ± 0.06	0.38 ± 0.06	0.54 ± 0.06
Isobutyrate	0.09 ± 0.08	0.38 ± 0.06	0.34 ± 0.06	0.47 ± 0.06
Isovalerate	0.08 ± 0.08	0.40 ± 0.06	0.37 ± 0.06	0.50 ± 0.06
Semen volume	0.51 ± 0.08	0.04 ± 0.03	0.06 ± 0.04	0.06 ± 0.04
Sperm concentration	0.49 ± 0.10	0.06 ± 0.04	0.08 ± 0.05	0.11 ± 0.06
Sperm motility	0.19 ± 0.11	1.72E−07 ± 0.02	1.23E−04 ± 0.03	4.40E−07 ± 0.04
Abnormal sperm rate	0.46 ± 0.10	4.15E−07 ± 0.02	3.61E−03 ± 0.03	2.43E−07 ± 0.03

${\mathbf{m}}_{\mathbf{h}}^{2}$: heritable microbes **m**^**2**^; ${\mathbf{m}}_{\mathbf{n}\mathbf{o}\mathbf{n}}^{2}$: non-heritable microbes **m**^**2**^. The number of heritable ASVs was 352, and the number of non-heritable ASVs was 675. The average microbiability can be calculated by dividing ${\mathbf{m}}_{\mathbf{h}}^{2}$ and ${\mathbf{m}}_{\mathbf{n}\mathbf{o}\mathbf{n}}^{2}$ by the corresponding number of ASVs

### Mediation analysis uncovers causal relationships between heritable genera and semen quality traits

To investigate whether microbiota influences host phenotypes via metabolic mediation, we conducted a mediation analysis. We initially conducted Spearman correlation analysis on the relative abundance of the 39 heritable genera and four semen quality traits, excluding 12 genera that did not exhibit significant associations (see Additional file 3: Figure S2). Next, we performed 648 mediation analyses involving the remaining 27 genera, six SCFAs parameters, and four semen quality traits. This analysis identified 99 significant mediation pathways linking genera, SCFAs, and semen quality traits (*P* < 0.05). Specifically, 44 genera significantly influenced sperm concentration through three SCFAs, 36 genera significantly influenced sperm motility through five SCFAs, and 19 genera significantly influenced abnormal sperm rate through three SCFAs (see Additional file 1: Table S4). Heritable microbiota had a more pronounced impact on abnormal sperm rate than on sperm motility (Table 1).

**Fig. 5:**
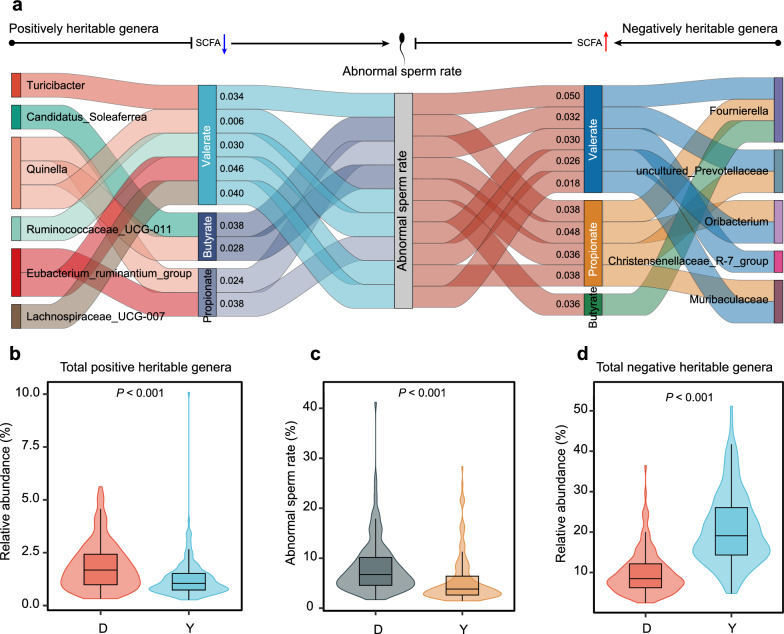
Relative abundance of heritable gut microbiota is linked to abnormal sperm rate in boars. **a** Mediation pathways among genera with heritable relative abundance, SCFAs, and abnormal sperm rate. Heritable genera either increase (left) or decrease (right) the abnormal sperm rate by altering propionate, butyrate, and valerate levels. *P*-values for mediated effects analysis are displayed next to each SCFA. **b** The total relative abundance of heritable bacteria that were positively correlated with abnormal sperm rate in the gut of Duroc and Yorkshire pigs. **c** Comparison of abnormal sperm rate between Duroc and Yorkshire boars. **d** The total relative abundance of heritable bacteria that were negatively correlated with abnormal sperm rate in the gut of Duroc and Yorkshire pigs. The Wilcoxon test was used for comparisons. *D* Duroc, *L* Landrace, *Y* Yorkshire

Figure 5a illustrates the mediation analysis results concerning abnormal sperm rate. A Sankey diagram illustrating how six heritable genera increase the abnormal sperm rate by inhibiting the production of three SCFAs (propionate, butyrate, and valerate). For instance, *Quinella* may elevate the abnormal sperm rate by reducing gut propionate (*P* = 0.024, *β* = 0.140), butyrate (*P* = 0.028, *β* = 0.145), and valerate (*P* = 0.006, *β* = 0.225) concentrations. Moreover, Fig. 5a shows that five heritable genera may lower the abnormal sperm rate by enhancing SCFAs production. For example, *Muribaculaceae* may counteract the rise in abnormal sperm rate by increasing gut propionate (*P* = 0.038, *β* = 0.344) and valerate (*P* = 0.018, *β* = 0.324) levels.

Duroc and Yorkshire boars, which are distantly related (Fig. 2d), showed the largest divergence in gut microbiota composition (Fig. 2a). A comparison of these two breeds revealed a significantly lower abnormal sperm rate in Yorkshire boars (*P* < 0.001, Fig. 5c). Further analysis showed that the total abundance of bacteria positively correlated with the abnormal sperm rate was higher in Duroc than in Yorkshire boars (*P* < 0.001, Fig. 5b), while the total abundance of bacteria that were negatively correlated with the abnormal sperm rate was lower in Duroc boars (*P* < 0.001, Fig. 5d). In summary, these findings suggest that variations in the relative abundance of heritable microbiota may affect boar semen quality phenotypes by regulating host SCFAs metabolism.

## Discussion

Recent studies indicate that the abundance of certain gut bacteria in pigs is influenced by host genetics [12, 13]. However, it remains unclear whether these heritable gut microbes mediate the influence of host genetics on boar semen quality phenotypes. In this study, using a population of 552 boars, we identified breed as a key determinant of gut microbiota composition, characterized heritable gut microbes, and analyzed the impact of heritable and non-heritable bacteria on SCFAs levels and semen quality traits. We also investigated the potential causal relationships between the abundance of heritable microbes, SCFAs, and semen quality, which may have significant implications for regulating boar semen quality.

In the present study, significant differences in semen quality were observed between boars from the three breeds, although they were reared under the same environmental conditions. Compared to Landrace and Yorkshire boars, Duroc boars exhibited lower semen volume, higher sperm concentration, and an elevated abnormal sperm rate, consistent with previous reports [27, 28]. This suggests that the influence of host genetics on boar semen quality is substantial and stable. While genetic variation is widely recognized as the primary driver of phenotypic differences, recent research suggests that variations in gut microbiota may also contribute to phenotypic differences among individuals within a population [9, 29]. Just as host genetics influences semen phenotypes, it also affects gut microbiota composition, with different boar breeds showing segregation in β diversity [9, 30, 31]. This raises the question whether part of the genetic influence on semen phenotypes is mediated by the abundance of heritable bacteria. In fact, several studies have revealed that the abundance of certain gut microbes is influenced by host genetics, ultimately contributing to alterations in host phenotypes [13, 32, 33].

Previous studies have demonstrated that closer genetic relationships among hosts correlate with more similar microbial compositions [13, 33]. Our study similarly found that the less related breeds exhibited more divergent microbial compositions. This indicates that host genetics influences microbial composition. Several studies have assessed the heritability of the gut microbiota in pigs across breeds [34], ages [35], and intestinal segments [13]. Although different experimental designs yielded slightly varying results, they consistently indicate the presence of highly heritable microorganisms in the pig gut. We found that the abundance of the Firmicutes phylum exhibits higher heritability than that of Bacteroidetes, which is consistent with previous studies [13, 35]. This may be due to the fact that Firmicutes bacteria themselves make up a much higher proportion of the porcine intestinal tract than Bacteroidetes, in addition to the fact that Bacteroidetes bacteria usually feed on carbohydrates and are, therefore, susceptible to environmental factors [36]. *Candidatus_Soleaferrea*, from the Firmicutes phylum, exhibited the highest heritability in this study, although there are limited and contradictory studies on the function of this bacterium. Some studies suggest an association with intestinal inflammation and metabolic diseases [37], while others propose a role in maintaining intestinal metabolic homeostasis [38, 39]. However, it is noteworthy that in several population-based Mendelian randomization studies, *Candidatus_Soleaferrea* has been used as an exposure factor and shown causal relationships with outcomes such as type 2 diabetes, autoimmune diseases, and gastroduodenal ulcer [37, 40, 41]. This suggests that, similar to our study, the abundance of *Candidatus_Soleaferrea* is also strongly associated with host genetics and phenotype in human populations.

Heritable and non-heritable microbes exhibited similar functions, with the most abundant functions involving carbohydrate metabolism, nucleotide synthesis, and amino acid and lipid synthesis. However, these functions are more enriched in heritable microbes, consistent with findings in dairy cows [25]. Carbohydrate metabolism primarily produces glucose, which supplies energy to the body. Ribulose-5-phosphate, an intermediate product of sugar metabolism, is extensively involved in nucleotide synthesis pathways [42]. Additionally, sugar metabolism provides key precursor molecules (e.g., acetyl-CoA, glycerol-3-phosphate) for lipid synthesis [43]. This establishes a close association between carbohydrate metabolism and both nucleotide metabolism and lipid synthesis pathways. Isoleucine and valine, essential amino acids for growth and metabolism in pigs, are key components of protein synthesis and are extensively involved in energy metabolism, immunomodulation, and intestinal homeostasis [44, 45]. It is noteworthy that some carbohydrates (e.g., dietary fiber and polysaccharides) cannot be directly digested and absorbed by the host but are fermented by gut microbes to produce SCFAs. In this study, we used fecal SCFAs as a proxy for microbial carbohydrate fermentation activity. This approach is based on the fact that most SCFAs produced in the gut are rapidly absorbed and metabolized by the host, making it difficult to directly quantify the total amount generated by gut microbes [46]. Therefore, under standardized sampling conditions, the fecal SCFA profile is still widely regarded as a practical indicator of gut microbial fermentation activity [47, 48]. SCFAs play a critical role in maintaining intestinal barrier function, regulating immune responses, and inhibiting the growth of pathogenic bacteria [3]. Many studies have shown that elevated SCFAs levels in the gut are associated with enhanced semen quality in pigs [6], chickens [49], and mice [7].

Our estimates of **h**^**2**^ and **m**^**2**^ for SCFAs and semen quality traits revealed contrasting patterns. Specifically, SCFAs exhibited low **h**^**2**^ but high **m**^**2**^, which is understandable given that SCFAs are fermentation end-products of dietary carbohydrates by gut microbes. Therefore, the microbial community composition plays a major role in determining SCFAs levels. In contrast, host genetic influence on SCFAs is likely indirect, potentially through its effects on absorption of SCFAs, metabolism, or regulation of gut physiology. On the other hand, semen quality traits showed higher **h**^**2**^ but lower **m**^**2**^. This reflects the polygenic nature of these traits, where host genetic variation can influence semen parameters through various biological pathways. Meanwhile, microbial effects on semen quality are likely indirect and context-dependent, making their direct influence more difficult to capture. Interestingly, when comparing microbiability attributed to heritable and non-heritable microbes, we found that, although non-heritable microbes contributed more to the overall **m**^**2**^ for most SCFAs and semen traits, the average **m**^**2**^ of heritable microbes per taxa was consistently higher than that of non-heritable ones. This finding highlights the substantial phenotypic impact that individual heritable microbes can exert [25]. For sperm motility and abnormal sperm rate, the **m**^**2**^ attributed to heritable microbes was nearly negligible. This could be due to the relatively low coefficients of variation for these traits or limitations in sample size that restricted power to detect significant microbial contributions. Alternatively, it is also possible that heritable microbes influence these traits indirectly, such as via SCFA-mediated pathways that modulate testicular or systemic physiological functions relevant to semen quality.

We further investigated whether SCFAs mediate the effect of heritable microbes on semen quality. We focused more on sperm motility and abnormal sperm rate, which reflect sperm quality, rather than semen volume and sperm concentration, which reflect sperm count. Although we found 36 significant mediating connections involving sperm motility, the sperm motility values were similar between breeds, and the microbiability of sperm motility was small. Thus, we shifted our focus to abnormal sperm rate. Heritable microbes primarily influenced abnormal sperm rate through the production of valerate, propionate, and butyrate. Heritable microbes that promoted SCFAs production were significantly enriched in the gut of Yorkshire boars, whereas those that inhibited SCFAs production were more abundant in Duroc boars. This partially explains the difference in abnormal sperm rates between the two pig breeds.

*Fournierella* [50]*, Prevotella* spp. [51] and *Muribaculaceae* [52], which are enriched in the gut of Yorkshire pigs, have been shown to ferment dietary fibers and produce abundant SCFAs. *Muribaculaceae*, in particular, is a primary fiber-degrading bacterium that breaks down complex fibers into smaller molecules for utilization by other bacteria [52]. In contrast, *Quinella*, which was enriched in the gut of Duroc pigs, is a secondary fiber-utilizing bacterium that can only use small carbohydrate molecules [53]. Additionally, *Turicibacter* has also been reported to be negatively correlated with the gut SCFAs content in pigs and may contribute to the development of chronic inflammation [54]. Thus, the abundance of heritable microbes may influence abnormal sperm rates in boars via SCFAs. As SCFAs play a crucial role in sperm redox homeostasis, they help maintain good sperm condition [49].

While this study identified heritable microbes in the boar gut and examined their contributions to semen quality, several limitations should be acknowledged. First, the relatively limited sample size may have constrained the statistical power to detect subtle host-microbe interactions and trait-specific associations. Increasing the sample size in future studies (particularly within a single breed) will likely enhance the ability to capture significant and biologically meaningful relationships between microbiota and reproductive traits. Additionally, this study used 16S rRNA sequencing, which restricts microbial resolution to the genus level. Future work incorporating metagenomic sequencing will allow species-level identification and functional profiling, providing deeper insights into the mechanisms by which heritable microbes affect host phenotypes.

## Conclusions

This study is the first to differentiate between heritable and non-heritable microbes in the boar gut, to analyze their microbiability on semen quality traits, and to establish a preliminary causal relationship between the abundance of SCFAs-mediated heritable microbes and semen quality. These findings enhance our understanding of the abundance of heritable microbes in the porcine gut and inform targeted regulation to improve boar semen quality.

## Supplementary Information

**Additional file 1: Table S1. **Composition and nutrient analysis of basal diet. **Table S2.** Estimates of the heritability of genera in the gut. **Table S3.** Estimates of the heritability of ASVs in the gut. **Table S4.** Inferences on the mediating effects of heritable genera, SCFAs, and semen quality traits.

**Additional file 2: Figure S1.** PCA of genetic structure in Duroc, Landrace, and Yorkshire boars.

**Additional file 3: Figure S2.** Heat map of Spearman correlations between 50 heritable bacteria and four semen quality traits.

## Data Availability

The 16S rRNA gene sequencing data are available from the Sequence Read Archive with accession number PRJNA1007937. The genotype files used in this study can be obtained through the figshare website, with a link of https://figshare.com/s/1b4b6203cceb3a20cdaa.
