# Supplementary figures and images for: Unraveling the role of bacteria with heritable versus non-heritable relative abundance in the gut on boar semen quality
Source: Genet Sel Evol. 2025 Nov 6;57:66. doi: 10.1186/s12711-025-00990-2 (PMC12590650; doi:10.1186/s12711-025-00990-2)

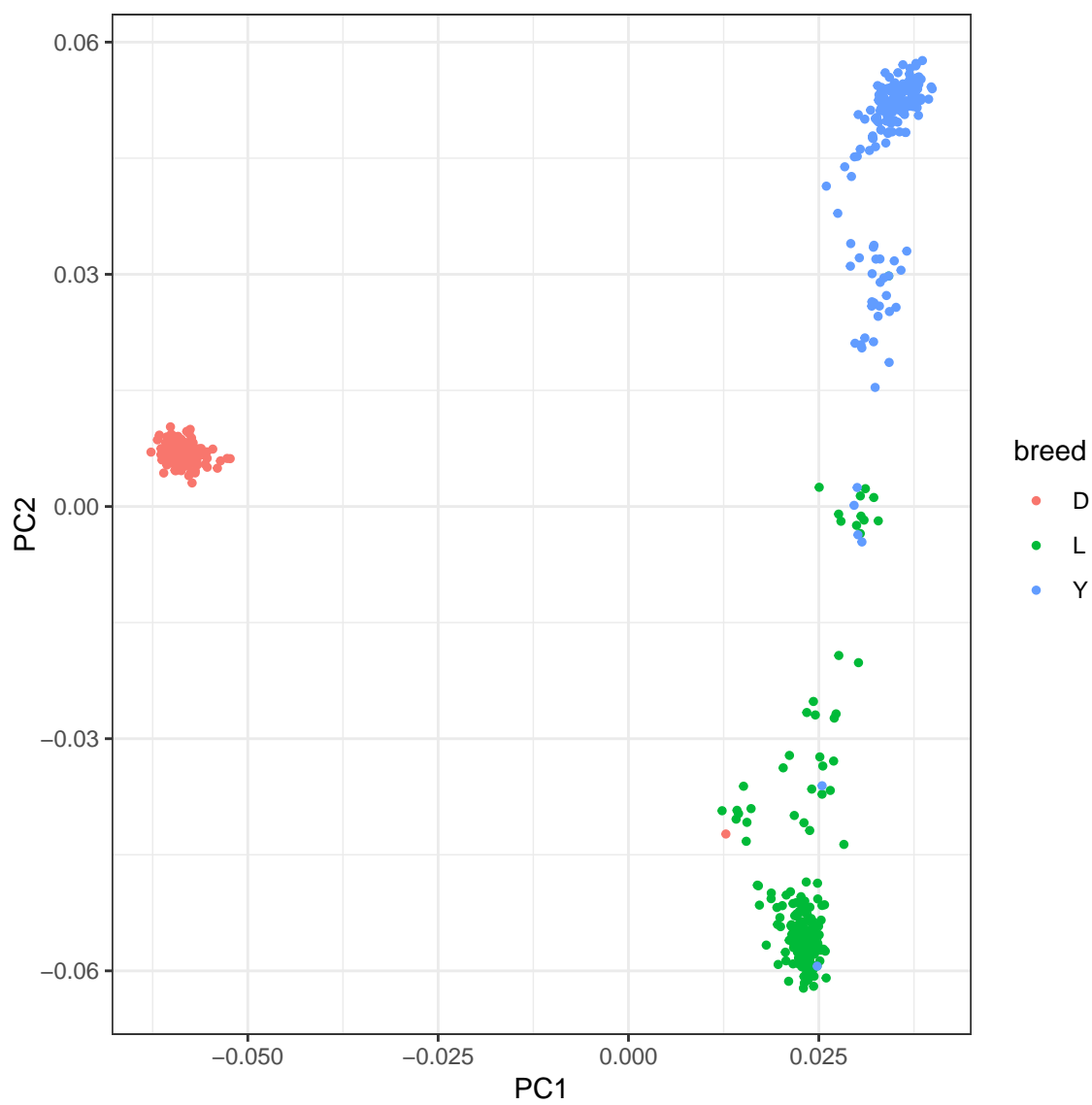

Supplement: Supplementary file 2 — Additional file 2: Figure S1. PCA of genetic structure in Duroc, Landrace, and Yorkshire boars. [file 12711_2025_990_MOESM2_ESM.pdf]

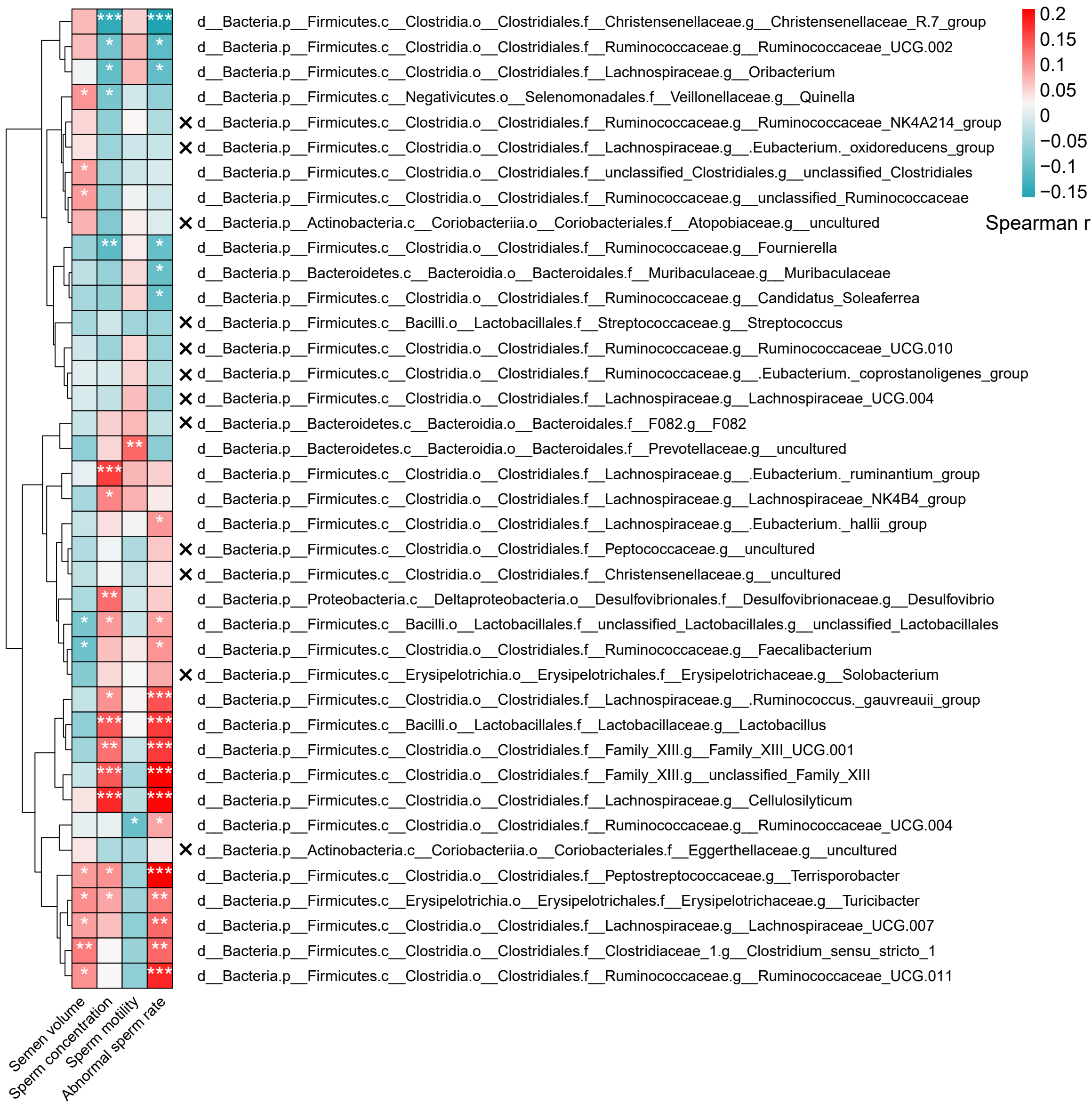

Supplement: Supplementary file 3 — Additional file 3: Figure S2. Heat map of Spearman correlations between 50 heritable bacteria and four semen quality traits. [file 12711_2025_990_MOESM3_ESM.pdf]
